# In regards to Pokhrel et al. Clinical validation of ring‐mounted halcyon linac for lung SBRT: Comparison to SBRT‐dedicated C‐arm linac treatments. JACMP 2021 Jan;22(1):261‐70

**DOI:** 10.1002/acm2.13273

**Published:** 2021-05-27

**Authors:** Biplab Sarkar, Tanmay Ghosh, Subhra Singhdha Biswal, Tanweer Shahid, Tharmarnadar Ganesh

**Affiliations:** ^1^ Department of Radiation Oncology Apollo Multispeciality Hospitals Kolkata India; ^2^ GLA University Mathura India; ^3^ Manipal Hospitals Dwarka India

## CONFLICT OF INTEREST

All authors have no conflict of interest to declare.

## AUTHORS CONTRIBUTIONS

Biplab Sarkar: Design, Data collection and writing; Tanmay Ghosh: Design, Data collection and writing; Subhra Singhdha Biswal: Design, Data collection and writing; Tanweer Sahid: Design, writing and Linguistic correction; Tharmarnadar Ganesh: Design, writing and Linguistic correction.

Dear Editor

This has reference to the above paper by Pokhrel et al., that compared stereotactic body radiotherapy (SBRT) plans for early stage I‐II‐ non‐small cell lung cancer done on two different linear accelerator (linac) systems: a C‐arm TrueBeam and a ring‐gantry type Halcyon (HA) (Varian Medical Systems, Palo Alto CA).^1^ We have a similar setup with one C‐arm Novalis Tx (NTX) and one ring‐gantry type Halcyon linac. Pokhrel et al., claimed that in their study they observed no significant difference in the organ at risk (OAR) doses between the two sets of plans and tolerance doses for all the OARs were achieved as per the Radiation Therapy Oncology Group (RTOG) protocol in both sets of plans.^1^, ^2^ Although this was true for majority of the similar lung SBRT cases in our setup, it was not always possible to comply with all RTOG‐0618 dose constraints for OARs for complex target volumes.^2^, ^3^ We found that in two out of nine cases, dose constraints were not achieved for one or more OARs in the HA plans as against the fully‐compliant NTX plans.^3^ In the HA plans, the dose constraint of V_20__Gy_ <15% was not achieved for the normal lung volume (lung‐planning target volume [PTV]) in both the cases, while in one case it was not possible to meet with the dose constraint for spinal cord (maximum dose <28 Gy). As shown in the plan comparison between NTX (triangles) and HA (squares) plans in Supplementary Figure 1, for a PTV‐prescription of 60 Gy in 5 fractions, with the same PTV‐coverage of 98% volume receiving 97% of prescription dose, the global dose maximums are 106.6% and 110.1% respectively; V_20__Gy_ for normal lung are 14.9% and 19.7% respectively. The HA plan failed to meet the OAR criteria of V20 Gy for the normal lung (Lung‐PTV) as per RTOG protocol 0618. ^3^ In NTX plans, approximately 40% were non‐coplanar beams, but in HA plans all beams were of coplanar geometry.

The delivery mechanisms and parameters differ substantially between NTX and HA systems. Beam characteristic of HA and NTX are unflatten and flatten respectively. The multileaf collimator (MLC) in NTX consists of 120 leaves (60 pairs), which includes 32 pairs of 2.5‐mm inner leaves and 28 pairs of 5‐mm outer leaves, while in HA the MLCs are of 10‐mm thick arranged in a dual, stacked layer to give an effective MLC thickness of 5 mm. The gantry speeds also differ — in NTX it is one revolution per minute (RPM) and in HA it is 2 RPM. The MLC speeds are also different between the systems with the NTX’s MLC speed slower at 2.5 cm/sec compared to HA’s 5.0 cm/sec. The maximum possible dose rates are 600 MU/min and 800 MU/min, respectively, for the NTX and HA systems. Such substantially differing critical delivery parameters will obviously result in different degrees of fluence modulation in these delivery systems which is evident from the huge difference in monitoring units (MU), viz., 6556.8 MUs for NTX vs. 3696 MUs for HA. With comparatively much lower MUs, it is expected that HA should result in a lesser low‐dose volume than NTX. However, as seen in Supplementary Figure 2, while the 50% isodose volumes are comparable for the two techniques (582 cc for NTX and 588.6 cc for HA), the 20% isodose volume for NTX (2394 cc) is 132 cc lesser than that for HA (2526 cc) even though the MUs for NTX is much higher. HA delivery technique results in 5.5% higher low‐dose region, and hence a higher dose to the large OARs, which in the present case is the lung‐PTV volume. Such a higher low‐dose region (V_20__Gy_ of lung‐PTV) in the HA technique is the result of the coplanar geometry employed in treatment delivery. Although in the thorax region there is limited scope for using non‐coplanar beams geometry due to patient‐couch‐gantry collision possibilities, it still has a vital role in treatment planning, especially when a tighter low‐dose region is required. Several groups have tried multiple non‐coplanar beam arrangements for SBRT on different sites like lung or liver.^4^ This technique is often associated with a mathematically incorrect terminology, namely “*4π geometry*” — which in reality uses a limiting 2π [$\lim_{\Omega \to 2\pi }\Omega$ where Ω is the solid angle] geometry beam‐arrangement approach to obtain a larger solid angle at the target ^4^, ^5^, ^6^ which results in a better dose distribution compared to that of a conventional coplanar beam geometry. ^4^

Even though the differences in delivery parameters like unflatten beam, higher MLC speed, higher gantry speed, and higher dose rate are in its favor, the Halcyon planning technique is seriously limited by the lack of non‐coplanar beam geometry which results in a larger low‐dose volume. If the vendor can incorporate a small yaw rotation to the couch (≈ ±10°‐± 15°) or a ± 10° gantry tilt against its rotational plane (as is possible in any modern CT‐scan machine), it will result in a better dosimetric outcome.

## Supporting information


**Supplementary Fig S1**. Comparison of radiotherapy treatment plans and dose distribution between Novalis Tx and Halcyon linear accelerators. Comparative dose‐volume histogram shows, for same target coverage, Halcyon produces a higher global dose maximum and the dose to lung‐PTV volume is in excess of the RTOG specified dose. Novalis Tx plan could meet dose constraints for all the OARs.

**Supplementary Fig S2**. Comparative analysis of the low‐dose region (volume receiving 50% and 20% of the prescription dose) for Halcyon and Novalis Tx plans. With 46% less MU, Halcyon plans contribute 5.5% additional 20% dose‐volume while for 50% isodose volume the plans are comparable.

## Data Availability

All data available with the corresponding author and can be produced as and when required.
