# Supplementary figures and images for: In regards to Pokhrel et al. Clinical validation of ring‐mounted halcyon linac for lung SBRT: Comparison to SBRT‐dedicated C‐arm linac treatments. JACMP 2021 Jan;22(1):261‐70
Source: J Appl Clin Med Phys. 2021 May 27;22(6):281–2. doi: 10.1002/acm2.13273 (PMC8200508; doi:10.1002/acm2.13273)

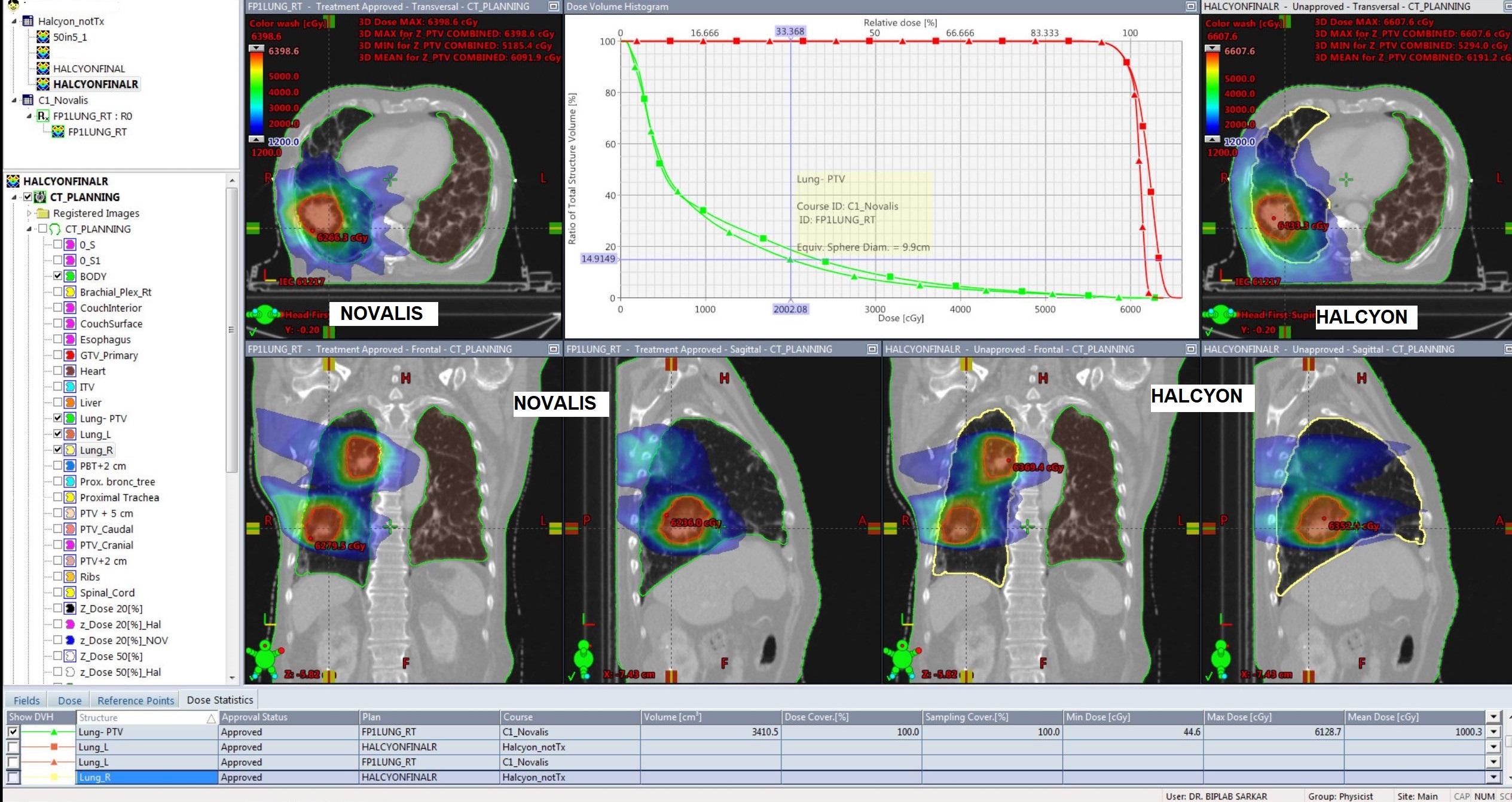

Supplement: Supplementary file 1 — Supplementary Fig S1. Comparison of radiotherapy treatment plans and dose distribution between Novalis Tx and Halcyon linear accelerators. Comparative dose‐volume histogram shows, for same target coverage, Halcyon produces a higher global dose maximum and the dose to lung‐PTV volume is in excess of the RTOG specified dose. Novalis Tx plan could meet dose constraints for all the OARs. [file ACM2-22-281-s002.jpg]

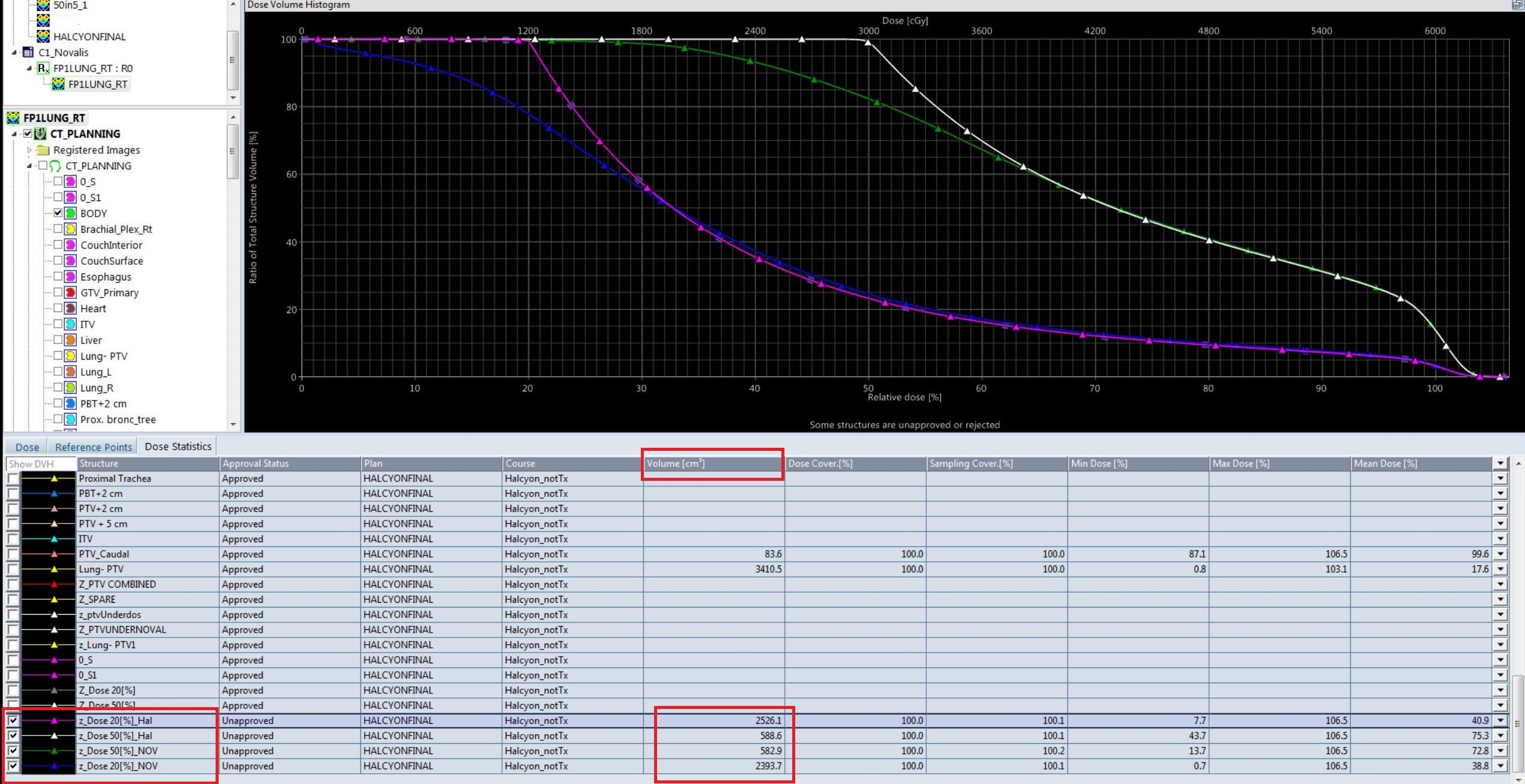

Supplement: Supplementary file 2 — Supplementary Fig S2. Comparative analysis of the low‐dose region (volume receiving 50% and 20% of the prescription dose) for Halcyon and Novalis Tx plans. With 46% less MU, Halcyon plans contribute 5.5% additional 20% dose‐volume while for 50% isodose volume the plans are comparable. [file ACM2-22-281-s001.jpg]
